# Expression of eicosanoid receptors subtypes and eosinophilic inflammation: implication on chronic rhinosinusitis

**DOI:** 10.1186/1465-9921-7-75

**Published:** 2006-05-12

**Authors:** Claudina Angela Pérez-Novo, Cindy Claeys, Paul Van Cauwenberge, Claus Bachert

**Affiliations:** 1 Upper Airways Research Laboratory, Department of Otorhinolaryngology, Ghent University Hospital, De Pintelaan 85, Ghent, Belgium

## Abstract

**Background:**

Eicosanoid receptors are G-protein-coupled receptors playing an important immunomodulatory role in airway diseases. However, there is little information on the expression of these receptors and their link with eosinophilic inflammation in paranasal sinus diseases. We aimed with this study to investigate the tissue expression of leukotrienes and prostaglandin E2 receptors in chronic rhinosinusitis patients and the link of this regulation with eosinophilic inflammation.

**Methods:**

Samples were prepared from nasal tissue of patients with chronic rhinosinusitis without nasal polyps (CRS, n = 11), with nasal polyps (CRS-NP, n = 13) and healthy subjects (Controls, n = 6). mRNA expression of CysLT_1_, CysLT_2_, BLT_1_, BLT_2_, E-prostanoid receptors (EP_1_, EP_2_, EP_3_, EP_4_) and sol-IL-5Rα was determined by real-time PCR. Concentrations of PGE2, LTC4/D4/E4, LTB4 and sol-IL-5Rα were determined by ELISA and of ECP by ImmunoCap. Protein expression and tissue localization of eicosanoid receptors and activated eosinophils were evaluated by immunohistochemistry.

**Results:**

CysLT_1 _mRNA expression was significantly increased in CRS-NP compared to CRS and controls, and CRS compared to controls, whereas CysLT_2 _mRNA was enhanced in both CRS groups without differences between them. Levels of both receptors correlated to the number of activated eosinophils, sol-IL-5Rα, ECP and LTC_4_/D_4_/E_4 _concentrations in the disease groups. PGE_2 _protein concentrations and prostanoid receptors EP_1 _and EP_3 _were down-regulated in the CRS-NP tissue vs. CRS and controls, whereas EP_2 _and EP_4 _expression was enhanced in CRS and CRS-NP patients vs. controls. No differences in BLT receptors were observed between patients and controls.

**Conclusion:**

CyLTs receptors are up-regulated in nasal polyp tissue and their expression correlate with eosinophilic inflammation supporting previous results. Eicosanoid receptors mRNA pattern observed suggests that down-regulation of EP_1 _and EP_3 _in CRS-NP and up-regulation EP_2 _and EP_4 _in CRS and CRS-NP groups may have some role in the development of the diseases and their regulation may not be directly linked to eosinophil activation but involve post-transcriptional events mainly related to other inflammatory cell sources.

## Background

The role of eicosanoids in the pathophysiology of chronic inflammatory airway diseases has been well documented; however, the key steps in the regulation leading to the production of these molecules remain unclear. Eicosanoid signalling pathway operates through lipid G-protein-coupled receptors (GPCRs) [1]. According to the International Union of Pharmacology (IUPHAR), eicosanoid receptors are classified in four main groups: the BLT receptors, with biological activities related to LTB_4_, the cysteinyl leukotrienes (cysLTs) receptors related family, the lipoxin (ALX) receptors and the prostanoid receptors class [1].

Interaction of cysteinyl leukotrienes receptors (CysLT_1 _and CysLT_2_) with theirs ligand LTC_4_, LTD_4 _and LTE_4 _play a disease-regulating role in chronic rhinosinusitis/nasal polyposis and particularly in the aspirin intolerance syndrome which is often correlated to these diseases. The biological actions of these molecules include endothelial cell adherence, myofibroblast proliferation, bronchoconstriction, vascular hyper-permeability, mucus secretion and chemokine production [2]. Immunohistochemical studies have revealed that the cysLTs receptors are expressed on eosinophils, mast cells, macrophages, neutrophils and vascular endothelial cells in the nasal mucosa [3]. Expression of these receptors has been also demonstrated in inflammatory cells in patients with seasonal allergic rhinitis [4]. In addition, *in vitro *studies involving cysLTs receptor antagonists have also demonstrated the crucial role of these molecules in the regulation of plasma extravasation and vascular endothelial growth factor synthesis [5]. These, are important events involved in the development of oedema formation in nasal polyps and in the pathogenesis of allergen-induced asthma. Of interest, a recent study performed in patients with chronic rhinosinusitis/aspirin intolerance showed that effects of cysteinyl leukotrienes in the nasal mucosa of these patients seems to occur mainly via interaction with CysLT_1 _on inflammatory leukocytes. However, effects of these eicosanoids on glands and epithelium may be mediated predominantly through CysLT_2 _[6].

Additionally, controversial results about LTB_4 _and BLT receptors have been reported in several studies. BLT_1 _is expressed primarily in leukocytes, human BLT_2 _is present in most human tissues [7,8]. In human peripheral blood leukocytes, neutrophils and eosinophils express significant amounts of both BLT_1 _and BLT_2_, whereas mononuclear cells express BLT_2 _but minimal BLT_1 _[8]. Expression of BLT receptors can be up-regulated during inflammation; however, the specific inflammatory stimuli responsible for their induction have not yet been fully characterized. In human airways, BLT receptor-LTB_4 _mediated action play a crucial role in host defence by regulating processes like recruitment, activation and survival of cells during inflammation [9-11]. However, until now, no clear mechanism regulating the synthesis of these molecules in airway has been yet demonstrated.

Finally, the role of prostaglandins in physiology and immune system is determined by multiple factors such as cellular context, receptor expression profile, receptor-ligand affinity and differential regulation of signal transduction pathways [12]. The prostanoid receptor subfamily comprises eight members: the prostaglandin D (DP) receptor, the prostaglandin E_2 _receptors (EP_1_, EP_2_, EP_3 _and EP_4_), the prostaglandin F receptor (FP), the prostaglandin I receptor (IP), the tromboxane A receptor (TP), and a ninth prostaglandin receptor identified recently, the chemoattractant receptor homologous molecule expressed on Th_2 _cells (CRTH2) [12].

In airways, PGE_2 _may induce bronchodilation and airway relaxation by acting via EP_2 _receptor [13,14]. Basal expression of EP_2 _and EP_4 _receptors is increased on bronchial inflammatory cells from asthmatic patients and may be altered *in vitro *on eosinophils in response to inflammatory stimuli, suggesting the immunomodulatory role of these receptors in asthma, [15]. More recently, a study comparing EP receptor expression in nasal biopsies from aspirin intolerant and tolerant patients showed an up-regulation of EP_1 _and EP_2 _in structural cells from aspirin intolerant subjects [15]. However, in the same study the number of inflammatory cells expressing EP_2 _but not EP_1_, EP_3 _or EP_4 _receptors was significantly up-regulated in the aspirin intolerant group [15].

Based on the previous studies, we hypothesize that eicosanoid receptor expression is altered in chronic rhinosinusitis patients with and without nasal polyposis in absence of aspirin intolerance and these changes may be related to eosinophilic inflammation.

## Methods

### Patients and clinical diagnosis

Samples from ethmoidal and maxillary sinuses were collected during functional endoscopic sinus surgery (FESS) procedures in the Department of Otorhinolaryngology at the Ghent University Hospital. Nasal tissues were obtained from 13 patients with chronic rhinosinusitis and nasal polyposis (CRS-NP) (10 males, 3 females, age range: 30–54 years) and 11 subjects with chronic rhinosinusitis without nasal polyposis CRS (8 males, 3 females, age range: 21–53 years) who were scheduled for sinus surgery in the department of Othorinolaryngology of the Ghent University Hospital. As control group (Controls), we included 6 subjects (4 males, 2 females, age range: 21–53 years), who underwent septal surgery and removal of parts of the inferior turbinate due to anatomical variations. These patients had any acute or chronic clinical, endoscopic or imaging signs of sinusitis or polyposis; and they did not show any history of atopic, or lower airway disease.

Diagnosis of CRS was based on the presence of typical symptoms (headache, nasal obstruction and discoloured nasal drainage) longer than 12 weeks and a positive CT-Scan showing swelling of the ethmoidal and maxillary mucosa and bilateral obstruction of the osteomeatal complex but without polyp formation, visible during nasal endoscopy or during surgery. Nasal polyposis was diagnosed based on symptoms history (nasal congestion, lost of smell, changes in sense of taste and persistent postnasal drainage), clinical examination, nasal endoscopy and paranasal sinuses CT-Scan, defined as presence of visible bilateral polyps growing from the middle meatus into the nasal cavities, and affecting the ethmoidal sinuses. In the CRS-NP group, three patients had asthma and in the CRS group, there was one patient with allergic rhinitis and one with asthma. Diagnosis of asthma was based on clinical history, typical symptoms and lung (pulmonary) function tests (Spirometry and Peak Expiratory Flow), following the Global initiative for asthma (GINA) guidelines. All patients have taken aspirin or other NSAIDs without manifesting any hypersensitive reaction.

The study was approved by the ethical committee of the Ghent University Hospital, and all patients gave informed consent before their participation. The use of any oral medication with possible impact on measurements of enzymes or mediators, including systemic glucocorticoids and anti-leukotrienes, was stopped in all subjects 4 weeks before surgery. The use of topical glucocorticoids was interrupted 2 weeks before surgery.

### Real time PCR for eicosanoid receptors

Quantitative real time PCR was used to determine the mRNA levels of eicosanoid receptors. Nasal tissue (30 mg) was homogenized in Tri-reagent buffer (Sigma-Aldrich, MO, USA), 1 ml per 50–100 mg of tissue, in a chilled pestle mortar. Total RNA from homogenates was isolated using Tri-reagent Kit following the manufacturer's instructions (Sigma-Aldrich, MO, USA). cDNA was synthesized from 2 μg of total RNA using Oligo(dT)_12–18_, random hexamers and the Superscript RNase H^-^Reverse Transcriptase (Life Technologies, CA, USA), as described previously [16]. Amplification reactions were performed on an iCycler iQ Real-Time PCR Detection System (Bio-Rad laboratories, CA, USA) using specific primers (Table 1), designed with the Primer3 software [17]. PCR reactions contained 20 ng cDNA (total RNA equivalent) of unknown samples, 1X SYBR Green I Master mix (Bio-Rad laboratories, CA, USA) and 300 nM of primer pairs in a final volume of 25 μl. PCR protocol consisted of 1 cycle at 95°C for 10 minutes followed by 45 cycles at 95°C for 30 seconds and at 60°C for 1 minute. The expression of two housekeeping genes Beta actin (ACTB) and Hydroxymethyl-bilane synthase (HMBS) was used to normalize for transcription and amplification variations among samples. The relative number of molecules of each gene, expressed in relative expression units quantified per 20 ng of cDNA sample, was determined by the *ΔCT *value method as described previously [18].

**Table 1:** Primer sequences used for real time PCR amplification

**Eicosanoid receptors**	**Forward primer (5'→ 3')**	**Reverse primer (5'→ 3')**	**Amplicon size**	**Genbank Accession number**
EP_1_	GATGGTGGGCCAGCTTGTC	GCCACCAACACCAGCATTG	73 bp	NM_000955
EP_2_	GACCGCTTACCTGCAGCTGTAC	TGAAGTTGCAGGCGAGCA	73 bp	NM_000956
EP_3_	AAGGCCACGGCATCTCAGT	TGATCCCCATAAGCTGAATGG	76 bp	NM_000957
EP_4_	ACGCCGCCTACTCCTACATG	AGAGGACGGTGGCGAGAAT	63 bp	NM_000958
BLT_1_	CCTGAAAAGGTGCAGAAGC	AAAAAGGGAGCAGTGAGCAA	93 bp	NM_000752
BLT_2_	CTTCTCATCGGGCATCACAG	ATCCTTCTGGGCCTACAGGT	88 bp	NM_019839
CysLT_1_	TCCTTAGAATGCAGAAGTCCGTG	AAATATAGGAGAGGGTCAAAGCAA	80 bp	NM_006639
CysLT_2_	GCTGATCATTCGGGTTCTGT	GGTGATGATGATGGTGGTCA	91 bp	NM_020377

### Eicosanoid levels

Concentration of cysteinyl leukotrienes C_4_/D_4_/E_4 _(LTC_4_/D_4_/E_4_), prostaglandin E_2 _(PGE_2_) and leukotriene B_4 _(LTB_4_) were measured by Enzyme Linked Immunoassays (ELISAs) purchased from Oxford BioMedicals (Oxford, USA). Sample extraction procedures for protein removal and eicosanoid stabilization were performed according to the provider's instructions. Briefly, nasal or sinus tissues were first homogenized in ethanol (5 ml/g) for LTB_4 _and LTC_4_/D_4_/E_4_, and in 15% methanol/0.1 M sodium phosphate buffer, pH 7.5 for PGE_2 _measurements and then centrifuged for 5 minutes at 3,000 rpm at 4°C. Supernatants were diluted in water, pH 3.5 and following manufacturer's instructions (Oxford BioMedicals, Oxford, USA). The detection ranges for all assays were between 0.02–10 ng/ml. The sensitivity was of 0.2 ng/ml for all assays and the intra-and inter-assay coefficient of variation less than 10%.

### Eosinophil inflammatory markers

Soluble IL-5Rα isoform was quantified using a real time PCR, as described previously [16]. Briefly, a standard curve was constructed from a plasmid containing the cDNA sequence for this receptor isoform. A fragment of this plasmid was amplified with specific primers, purified and used in equimolar 10-fold dilutions to generate a standard curve. Real time amplifications were performed in a 25 μl volume reaction containing 1X SYBR Green I Master mix (Bio-Rad laboratories, CA, USA), 300 nM of primer pairs and a set of primers specific for this hIL-5Rα isoform [16]. PCR protocol consisted of 1 cycle at 95°C for 10 minutes followed by 40 cycles at 95°C for 30 seconds and at 64°C for 1 minute. Each sample was tested in duplicate. The quantity of each amplicon was calculated from the values of the standard curve and normalized by the quantities obtained for beta actin (ACTB) and hydroxymethyl-bilane synthase (HMBS).

Soluble IL-5α receptor protein concentrations were measured by a research ELISA as described previously [19] with a sensitivity of 8 pg/ml. and an intra-and inter-assay coefficient of variation less than 10%. Quantification of Eosinophil Cationic Protein (ECP) was carried out, on supernatants obtained after nasal tissue homogenization, by the UniCAP system (Pharmacia & Upjohn, Sweden), with a detection limit of < 0.5 μg/L and a coefficient of variation less than 10%.

The number of activated eosinophils was determined by staining the eosinophil granulocyte (EG_2_) and semiquantitative scoring of positively stained cells on the different tissues. For that, frozen tissue sections were fixed in acetone for 10 minutes, washed in TBS buffer and incubated with (1:1000) mouse anti-human ECP/EPX monoclonal antibody (Pharmacia & Upjohn Uppsala, Sweden) for 1 hour. Then, the slides were incubated with (1:50) rabbit anti-mouse IgG for 10 minutes and developed with (1:100) alkaline phosphatase anti-alkaline phosphatase (Dako, Glostrup, Denmark) for 10 minutes at room temperature. Signal detection was performed using the New Fuchsin Substrate System, following the manufacture's instructions (Dako, Glostrup, Denmark). Semiquantitative scoring was performed by a pathologist, who was blinded for the clinical data, on a four-point scale adapted from an already validated system of semiquantitative evaluation. Zero represented the lowest and three the highest score. The analysis included all areas of the biopsies and a global score was given for each parameter.

### Immunohistochemical staining for prostanoid and leukotriene receptors

Frozen tissue sections were fixed in acetone for 10 minutes at room temperature and washed in 1X TBS buffer. Endogenous peroxidase activity was blocked with 0.3 % hydrogen peroxidase (VWR International, Pennsylvania, USA) in PBS containing 0,001 % NaN_3 _for 20 minutes at room temperature. Sections were than washed for 10 minutes with 1X TBS and incubated with foetal bovine serum during 30 minutes. Sections were then incubated for 1 hour at room temperature with primary antibodies: rabbit IgG polyclonal Antibodies for EP_1 _receptor (1:250), EP_2 _receptor (1:250), EP_3 _receptor (1:200), EP_4 _receptor (1:250), CysLT_1 _(1:50) and CysLT_2 _(1:50), purchased by Cayman Chemicals (MI, USA). Signal was detected with LSAB^+ ^kit (HRP Rabbit/Mouse/Goat) purchased from Dako, using the AEC^+ ^High Sensitivity Substrate Chromogen Kit (Dako, Glostrup, Denmark). To control for unspecific binding of the primary antibodies used in the study, parallel stainings were performed omitting the primary antibody and by substituting it with an irrelevant antibody or non-immune sera of the same species (isotype), at the same concentration as the specific antibody (antisera).

### Statistical data analysis

All data was analyzed using the MedCalc software version 6.0 (Mariakerke, Belgium). Results are presented in Box-and-Whisker plots, where the central box represent the values from the lower and upper interquartile range, and the middle line the median. Data comparison within different patient groups was performed using the Kruskal-Wallis test (*H-test*). The Wilcoxon test (or *Mann-Whitney U test*) for unpaired samples was applied to evaluate the statistical differences between two patient groups. Spearman's rank correlation analysis was performed to determine statistical significance of differences between two parameters in a classification group. *P *values equal or less than 0.05 was regarded as significant.

## Results

### mRNA expression of eicosanoid receptors by real time PCR

**Figure 1:**
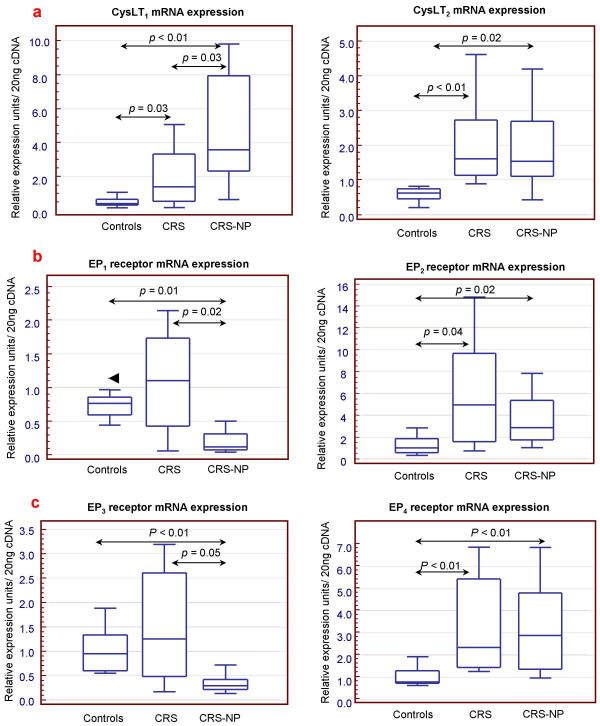
**mRNA levels of eicosanoid receptors in nasalmucosa**. **a) **cysLTs receptors, **b) **prostanoid-E receptors. *Controls: *healthy subjects, *CRS*: chronic rhinosinusitis, *CRS-NP: *chronic rhinosinusitis/nasal polyps. *P: p *value (unpaired *Mann-Whitney U *test).

Expression of leukotrienes and prostanoid receptors analyzed by quantitative real time PCR showed an up-regulation of CysLT_1 _and CysLT_2 _receptors in CRS patients compared to controls, but only CysLT_1 _was significantly higher in CRS-NP in comparison to CRS patients as showed in Figure 1a. EP_2 _and EP_4 _mRNA was up-regulated in both CRS groups when compared with normal subjects (Figure 1b). In contrast, EP_1 _and EP_3 _expression was similar in controls and CRS patients but significantly down-regulated in the CRS-NP group (Figure 1b). Concentrations of BLT_1 _and BLT_2 _receptors were similar in the three groups of patients (data not shown).

**Figure 2:**
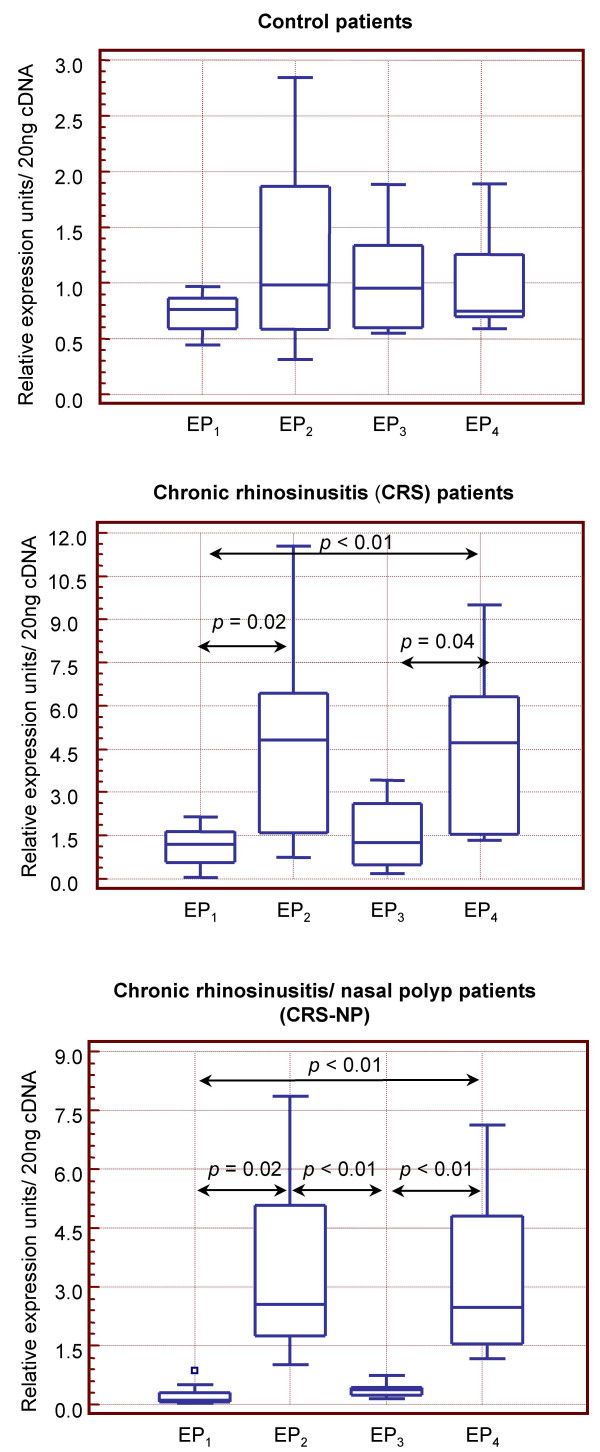
**Balance of mRNA levels of prostanoid E receptors innasal mucosa**. *Controls: *healthy subjects, *CRS*: chronic rhinosinusitis, *CRS-NP: *chronic rhinosinusitis/nasal polyps. *P: p *value (unpaired *Mann-Whitney U *test).

**Figure 3:**
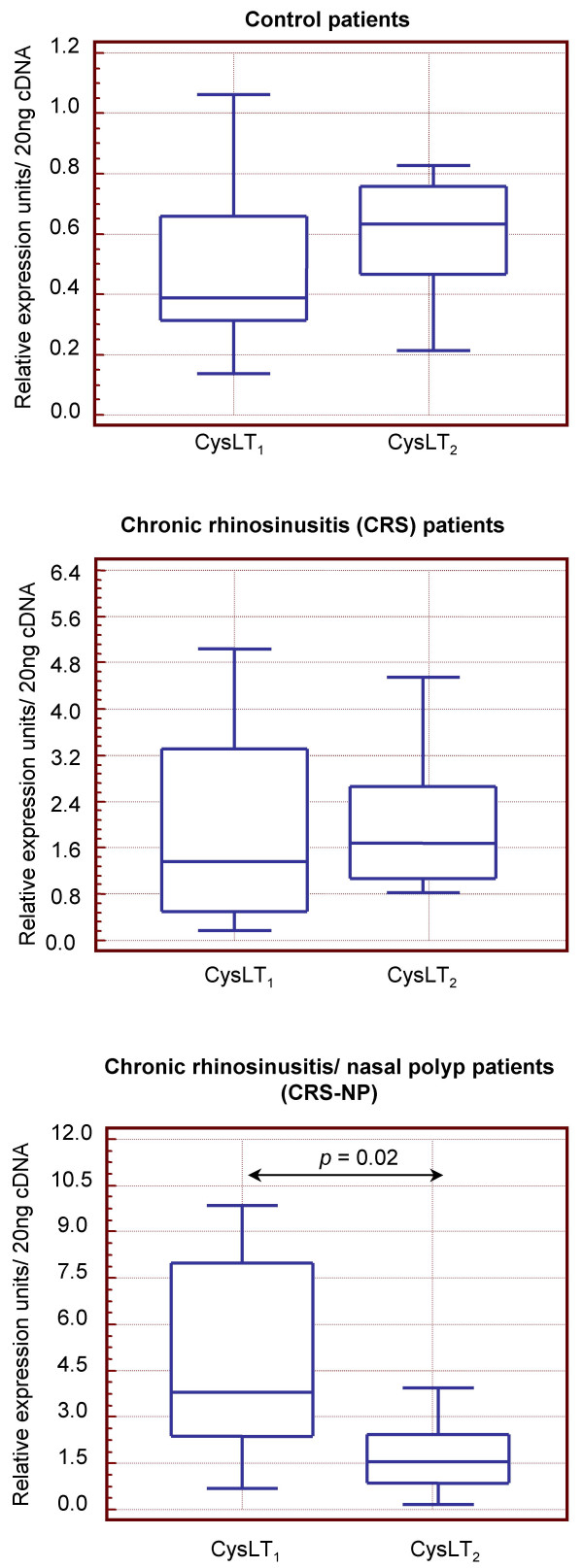
**Balance of mRNA levels of cysteinyl leukotriene receptors in nasal mucosa**. *Controls: *healthy subjects, *CRS*: chronic rhinosinusitis, *CRS-NP: *chronic rhinosinusitis/nasal polyps. *P: p *value (unpaired *Mann-Whitney U *test

Expression profile for the four EP and the two cysLTs receptors analyzed in each individual group of patients is illustrated in Figures 2 and 3. In the control group, expression of EP receptors did not show any differences. However, in the CRS and EP_1 _and EP_3 _receptors were down-expressed compared to EP_2 _and EP_4 _and this difference was more accentuated in the CRS-NP subjects. On the other hand CysLT_1 _and CysLT_2 _mRNA levels were similar in CRS and normal mucosa, however significantly higher concentrations of CysLT_1 _compared to CysLT_2 _were observed in the CRS-NP group.

### Eicosanoid levels and eosinophil inflammatory markers

Levels of eicosanoids and eosinophilic inflammatory markers are summarized in table 2. While concentrations of LTC_4_/D_4_/E_4_, were significantly higher in CRS-NP compared to CRS and controls, no differences were observed in LTB_4 _levels between the groups. PGE_2 _concentrations however, were similar in CRS and control (*p *> 0.10) but statistically lower in the nasal polyp tissue (*p *= 0.001). Real time PCR for sol-IL-5α R showed a significantly increase in CRS-NP subjects compared to CRS and in CRS compared to control subjects. Accordingly, sol-IL-5Rα protein was also statistically higher in the CRS-NP compared to CRS and in this group compared to control tissue. ECP was significantly increased in CRS compared to the control group (*p *< 0.05) and even more in CRS-NP (*p *< 0.02).

**Table 2:** Concentration of eicosanoids and eosinophilic inflammation markers in chronic rhinosinusitis patients

	**Controls**	**CRS**	**CRS-NP**	***Kruskall Wallis-test***
**Eicosanoids**				

LTC_4_/D_4_/E_4 _(ng/ml)	1.16 (IQR: 0.85–1.68)	3.34 (IQR: 2.70–5.35)	7.24 (IQR: 4.65–12.40)	*P *< 0.01 *§*
LTB_4 _(ng/ml)	25.25 (IQR: 8.26–63.91)	21.95 (IQR: 9.40–31.90)	19.44 (IQR: 12.80–29.71)	N.S
PGE_2 _(ng/ml)	180.63 (IQR: 101.44–258.86)	199.83 (IQR: 59.10–223.52)	55.00 (IQR: 40.59–67.87)	*p *= 0.02 ¶

**Eosinophilic inflammation markers**				

ECP (μg/L)	602.51 (IQR: 309.90–894.30)	2090.00 (IQR: 1437.60–5442.40)	11880.00 (IQR: 1862.70–17920.74)	*p *< 0.01 *§*
Sol-IL-5Rα protein (ng/ml)	20.62 (IQR: 15.77–26.43)	50.95 (IQR: 28.62–67.78)	175.24 (IQR: 37.11–309.67)	P < 0.05 *§*
sol-IL-5Rα mRNA (pg/μl)	14757.50 (IQR: 12493.97–23015.35)	159065.30 (IQR: 45909.00–185796.90)	458449.55 (IQR: 267447.00–796387.30)	*p *= 0.02 *§*
EG_2 _positive cells	1,00 (IQR: 1,00–1,15)	1,05 (IQR: 1,00–1,40)	2,10 (IQR: 1,90–2,25)	*p *< 0.01 ¶

Results are expressed as median and interquartile ranges (IQR). *§: p *< 0.05 is due to differences between the three sample groups; (¶): *p *< 0.05 is due to differences in the levels of CRS-NP versus controls and CRS patients. *N.S: *No statistical differences, *p *value > 0.05. *CRS: *chronic rhinosinusitis, *CRS-NP*: chronic rhinosinusitis/nasal polyp, *Controls*: healthy subjects.

Immunohistochemistry results demonstrated a strong infiltration of inflammatory cells in the nasal polyp compared to the CRS and inferior turbinate tissues (data not shown) and the median score for EG_2 _positive cells was significantly higher in NP tissue compared to the control and CRS tissues as summarized in Table 2.

The Spearman's rank correlation analysis showed a strong correlation between both cysLTs receptors mRNA with sol-IL-5Rα protein concentrations (CysLT_1_: *rho *= 0,574, *p *= 0.01; CysLT_2_: *rho *= 0,523; *p *< 0.05), ECP (CysLT_1_: *rho *= 0,544, *p *= 0.02; CysLT_2_: *rho *= 0,413; *p *= 0.03) and the total number of activated eosinophils (CysLT_1_: *rho *= 0,546, *p *= 0.02; CysLT_2_: *rho *= 0,614; *p *= 0.03). No correlations were found between the levels of prostanoid receptors and eosinophilic inflammation markers.

### Immunohistochemical staining for prostanoid and leukotriene receptors

**Figure 4:**
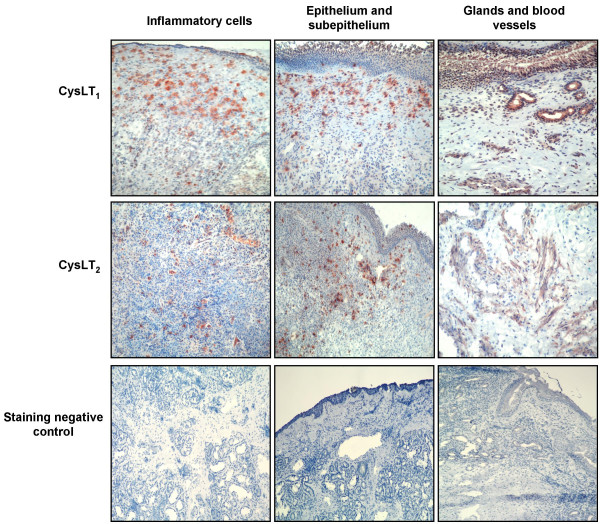
**Cysteinyl leukotriene receptors in chronicrhinosinusitis tissue**. Representative photomicrography (original magnification 40 ×) of chronic rhinosinusitis/nasal polyp specimens immunostained for CysLT_1 _and CysLT_2 _receptors in inflammatory cells, epithelium, glands and blood vessels.

Immunohistochemical staining for CysLT_1 _and CysLT_2 _receptors in the nasal tissue is represented in Figure 3. Immunoreactivity of both cysLTs receptors was observed in inflammatory cells in the lamina propria in both CRS groups. In addition, these receptors were expressed in the sub-epithelial layer of the nasal mucosa and to a lesser extend in the epithelium. Prostanoid-E receptors were mainly expressed in the epithelium and in mucosal glands (Figure 4). Immunoreactivity for EP_2 _and EP_4 _was higher in inflammatory cells compared to epithelium, contrary to EP_1 _and EP_3 _staining, which was localized mainly in epithelial and sub-epithelial regions and blood vessels.

## Discussion

Several studies have suggested changes in the eicosanoid regulation patterns as one of the factors involved in the pathophysiology of chronic rhinosinusitis and nasal polyposis; however, the effect of eicosanoids in the tissue, greatly dependents of the differential expression of the distinct subtypes of their receptors.

In this study, we confirmed that CysLT_1 _and CysLT_2 _receptors are up-regulated in chronic rhinosinusitis and nasal polyp patients. Interestingly, the balance of these receptors was similar in healthy and chronic rhinosinusitis subjects, in contrast to the nasal polyp group where expression of CysLT_1 _was significantly higher when compared to CysLT_2_. Furthermore, we evaluated the link between these receptors and eosinophilic inflammation and we observed that both CysLT_1 _and CysLT_2 _correlated with markers of eosinophil activation like IL-5Rα, ECP and the number of activated eosinophils. The data obtained are in line with previous results showing that eosinophils are one of the most important sources of these receptors in inflamed upper airways [20,21]; and that CysLT_1 _maybe involved in several stages of eosinophil differentiation, recruitment and maturation [22-24]. In other hand, we did not found any changes in BLT receptors expression between controls and disease groups. These findings correspond with previous studies performed in aspirin intolerant nasal polyp patients [25] and with perennial allergic rhinitis [26]. However are in contrast with other reports showing increased levels of LTB_4_ and BLT receptors  in allergic versus non-allergic nasal polyp patients [10]. Accordingly, there are no clear evidences about the role of these molecules in chronic rhinosinusitis and nasal polyposis and following our results, we question its role in these diseases.

As well as leukotrienes, prostaglandins and especially PGE_2 _play an important role in the regulation of the inflammatory process observed in chronic rhinosinusitis patients [27]. Little is known about the function and distribution of these receptors in airways and there are almost no studies reporting the action or regulation of these receptors in upper airway tissue. We show with this work that mRNA profile of prostanoid E receptors differs between chronic rhinosinusitis with and without polyps, again being different from healthy controls. We also observed that EP_2 _and EP_4 _receptors are up-regulated in chronic rhinosinusitis and nasal polyp tissue compared to control subjects; however, EP_1 _and EP_3 _transcripts were statistically decreased in the nasal polyp patients.

It has been reported, that action of agonists of EP_1_, EP_4 _and of a variant of EP_3 _is mediated by increase of intracellular cAMP [28,29]. In inflammatory cells, this phenomenon is associated with an inhibition of effector's cell functions such as activation, or response to certain stimulus [28,29]. Accordingly, we can assume that down-regulation of these receptors may be related to an increase of functionality or maybe susceptibility of inflammatory cells to pro-inflammatory stimulus like IL-5 and leukotrienes contributing to the chronic inflammation observed in chronic rhinosinusitis and even more in nasal polyposis. However, and in contrast with the previous statement, interaction of PGE_2 _with EP_1 _and EP_3 _variants is translated in an increase of intracellular calcium, which can results in an induction of immune cell activation [29]. This process is of great importance in chronic rhinosinusitis/nasal polyposis because increase of intracellular calcium may induce the activation of cytosolic phospholipase A_2 _leading to the production of leukotrienes and other pro-inflammatory lipid mediators again contributing to the chronic inflammatory process observed in these diseases. However, this is in contradiction with our results where EP1 and EP3 are down- regulated in the disease groups.

Furthermore, an *in vitro *study performed on inflammatory cells demonstrated that eosinophils express high levels of EP_2 _and EP_4 _mRNA in comparison with EP_1 _and EP_3_, which were almost not present in these cells and that deficiency of PGE_2 _production may up-regulate the expression of EP_2 _and EP_4 _molecules [30]. This is on line with our findings however, mRNA levels of EP_2 _and EP_4 _receptors did not correlate with eosinophil number or eosinophil activation markers but it was increased in the disease groups compared to controls. In addition, PGE_2 _was down-regulated in chronic rhinosinusitis/nasal polyp tissue compared to chronic rhinosinusitis and normal nasal mucosa and the levels of this eicosanoid inversely correlated to eosinophilic inflammation. Analysing closely these results one may suggest that although synthesis of PGE_2 _may be related to eosinophil activation, regulation of its receptors at least at mRNA levels depends of mechanisms involving other cellular sources as showed recently by Ying and col. [15]. In addition, the lack of correlation and the similar mRNA profile of these receptors between the disease groups also suggest that functionality of these receptors may greatly depend of post-transcriptional regulation mechanisms. Here we were not able to analyze the protein expression of these molecules to confirm our PCR results but studies performed in nasal polyp patients partially support this hypothesis [15].

## Conclusion

mRNA pattern of eicosanoid receptors is different between chronic rhinosinusitis and chronic rhinosinusitis/nasal polyp patients and compared to healthy subjects. CyLTs receptors are up-regulated in nasal polyp tissue and correlate with eosinophilic inflammation supporting previous results. Eicosanoid receptors mRNA pattern observed in our patient's groups suggest that down-regulation of EP_1 _and EP_3 _in the nasal polyp tissue and up-regulation EP_2 _and EP_4 _in both chronic rhinosinusitis groups may play a role in the development of the diseases and their regulation do not directly depend of eosinophil activation. Furthermore, these results also suggest the importance of post-transcriptional events in the regulation of receptor functionality involving other inflammatory multiple cellular sources. This is a descriptive preliminary study, which opens the door to more specific experiments including protein regulation, and functional studies that will reveal more information about the role of these receptors in chronic sinuses diseases.

## Abbreviations

BLT_1_: leukotriene B_4 _receptor 1

BLT_2_: leukotriene B_4 _receptor 2

CRS: chronic rhinosinusitis

CRS-NP: chronic rhinosinusitis/nasal polyp

CysLTs: cysteinyl leukotrienes

CysLT_1_: cysteinyl leukotriene receptor 1

CysLT_2_: cysteinyl leukotriene receptor 2

EG_2_: eosinophil granulocyte

EP_1_: prostanoid receptor 1

EP_2_: prostanoid receptor 2

EP_3_: prostanoid receptor 3

EP_4_: prostanoid receptor 4

Sol-IL5Rα : soluble interleukin -5 receptor alpha

LTC_4_/D_4_/E_4_: leukotriene C_4_/D_4_/E_4_

LTB_4_: leukotriene B_4_

PGE_2_: prostaglandin E_2_

## Competing interests

There are no "financial competing interests" or other "non-financial competing interests" involving political, personal, religious, ideological, academic, intellectual, commercial or any other to declare in relation to this manuscript.

## Authors' contributions

CAPN: Main person in the design of the study, designed the primers sequences and optimized the real time PCR protocols, performed the statistical analysis and wrote the manuscript.

CC: Collected and prepare the samples for the study, performed the measurements by ELISA and helped to draft the manuscript

PVC: participated in the design of the study and helped to draft the manuscript

CB: was involved in the design of the study and analysis of the results, helped to draft and revise the manuscript.

**Figure 5:**
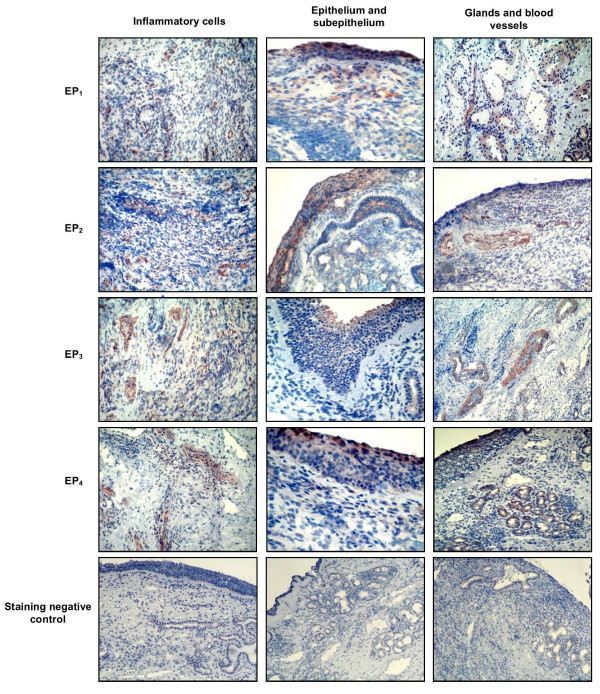
**Prostanoid E receptors in chronic rhinosinusitistissue**. Representative photomicrography (original magnification 40 ×) of chronic rhinosinusitis/nasal polyp specimens immunostained for EP_1_, EP_2_, EP_3 _and EP_4 _receptors in inflammatory cells, epithelium, glands and blood vessels.
